# Cognitive Load Does Not Affect the Behavioral and Cognitive Foundations of Social Cooperation

**DOI:** 10.3389/fpsyg.2016.01312

**Published:** 2016-08-31

**Authors:** Laura Mieth, Raoul Bell, Axel Buchner

**Affiliations:** Department of Experimental Psychology, Heinrich Heine University DüsseldorfDüsseldorf, Germany

**Keywords:** dual task, working memory load, trust, social cooperation, source memory

## Abstract

The present study serves to test whether the cognitive mechanisms underlying social cooperation are affected by cognitive load. Participants interacted with trustworthy-looking and untrustworthy-looking partners in a sequential Prisoner’s Dilemma Game. Facial trustworthiness was manipulated to stimulate expectations about the future behavior of the partners which were either violated or confirmed by the partners’ cheating or cooperation during the game. In a source memory test, participants were required to recognize the partners and to classify them as cheaters or cooperators. A multinomial model was used to disentangle item memory, source memory and guessing processes. We found an expectancy-congruent bias toward guessing that trustworthy-looking partners were more likely to be associated with cooperation than untrustworthy-looking partners. Source memory was enhanced for cheating that violated the participants’ positive expectations about trustworthy-looking partners. We were interested in whether or not this expectancy-violation effect—that helps to revise unjustified expectations about trustworthy-looking partners—depends on cognitive load induced via a secondary continuous reaction time task. Although this secondary task interfered with working memory processes in a validation study, both the expectancy-congruent guessing bias as well as the expectancy-violation effect were obtained with and without cognitive load. These findings support the hypothesis that the expectancy-violation effect is due to a simple mechanism that does not rely on demanding elaborative processes. We conclude that most cognitive mechanisms underlying social cooperation presumably operate automatically so that they remain unaffected by cognitive load.

## Introduction

There is increasing interest in whether (and how) social cooperation is affected by cognitive load. Although it has been proposed that cooperation is generally decreased (Piovesan and Wengström, 2009) or enhanced (Rand et al., 2012) by cognitive load, no consensus about this issue has been reached, and there are a number of null findings and failed replications (Tinghög et al., 2013; Kessler and Meier, 2014; Verkoeijen and Bouwmeester, 2014). Focusing on how cognitive load affects specific cognitive mechanisms that are important for cooperation could be a more promising approach than looking at the global outcome of presumably many different kinds of processes involved in cooperation. Therefore, the present study examines how memory for cheating or cooperation—a necessary prerequisite for reciprocal cooperation (Trivers, 1971)—is affected by cognitive load. We were particularly interested in whether or not social expectations affect the participants’ memory for the cheating or cooperation of interaction partners under cognitive load.

Examining the influence of social expectations seems particularly important because social cooperation depends fundamentally on expectations about other people’s behaviors. This can be illustrated with the Prisoner’s Dilemma Game (Clark and Sefton, 2001), which serves as a model for understanding human cooperation. In this game, two players independently decide whether or not to cooperate with each other. Mutual cooperation leads to reward while mutual defection leads to punishment, which reflects that more can be achieved through cooperation. However, unilateral defection leads to the highest payoff (the temptation payoff) while unilateral cooperation leads to the worst payoff (the sucker’s payoff). The dilemma lies in the fact that each player can maximize his or her payoff by defecting, but mutual defection leads to a worse payoff for both players than mutual cooperation. Humans are often able to resist the selfish temptation to defect, and high levels of cooperation are often achieved even in one-shot games (Delton et al., 2011). However, given that nobody wants to be suckered, cooperation depends on people’s expectations about whether or not the other player will choose to cooperate.

These expectations are strongly influenced by facial appearance (Chang et al., 2010; Olivola and Todorov, 2010). Appearance-based impressions are formed quickly (Willis and Todorov, 2006; Todorov et al., 2009) and automatically (Engell et al., 2007), but are quite stable over time. There is also a high degree of inter-individual agreement about who looks trustworthy and who does not (Todorov, 2008). These appearance-based impressions determine people’s behaviors in social-dilemma games: People often cooperate with trustworthy-looking partners, and defect against untrustworthy-looking partners (van ’t Wout and Sanfey, 2008; Rezlescu et al., 2012).

However, appearance-based expectations may often turn out to be false. People are somewhat better than chance when using facial appearance to predict whether partners will cooperate or cheat in social-dilemma games (Bonnefon et al., 2013), but facial appearance is a comparatively invalid source of information about a person’s character, and people rely on it more than they should (Olivola and Todorov, 2010). Therefore, remembering expectancy-incongruent information is especially important to correct invalid appearance-based impressions about other persons. To correct a false impression, it is insufficient to simply recognize the face as familiar, it is also necessary to have good source memory for the association between the face and the behavior of the person (Buchner et al., 2009). For example, remembering that a trustworthy-looking person is unreliable is important to avoid being misled by the person’s trustworthy appearance in the future. This functional analysis leads to the prediction that people should have better source memory for expectancy-incongruent information than for expectancy-congruent information.

The same prediction can be derived from schema theories of memory. The schema-copy-plus-tag model (Graesser and Nakamura, 1982) implies that expectancy-congruent behaviors are represented in memory by pointers to general schemas. Expectancy-violating behaviors are tagged as schema violations. In memory tests, participants often produce a high amount of schema-congruent information due to guessing, but memory accuracy is often poor for this type of information because it is produced regardless of whether it was present at encoding or not. The discrimination between actually experienced and new information is often better for schema-atypical information. For instance, participants will guess that a trustworthy-looking face belongs to a trustworthy person, regardless of whether the behavior of the person was trustworthy or not. Learning that a trustworthy-looking person is a cheater represents a more distinct and therefore more memorable information. Indeed, several studies confirmed the idea that people remember appearance-incongruent behaviors better than appearance-congruent behaviors (Suzuki and Suga, 2010; Volstorf et al., 2011; Bell et al., 2012b).

The present study serves to test whether or not the memory advantage for expectancy-incongruent behavior depends on cognitive load. Two opposing hypotheses are tested. Source memory for cheating and cooperation may be *impaired* by cognitive load because source memory is often believed to be more fragile and more dependent on cognitive resources than familiarity-based item memory (Nieznański, 2013). Therefore, the encoding of the association between a face and cheating or cooperation may be decreased under cognitive load. Memory for expectancy-incongruent information in particular may be negatively affected because this information cannot be easily integrated into existing schemas. Expectancy-incongruent information may trigger more effortful elaborative encoding than expectancy-congruent information, which will lead to enhanced memory for this information under normal circumstances. However, these elaborative processes may depend on the mobilization of additional cognitive resources. Therefore, a reduction in available cognitive resources may eliminate the expectancy-violation effect. Consistent with this hypothesis, the source memory advantage for expectancy-incongruent information was absent in older adults (Bell et al., 2013) who may have fewer cognitive resources available than younger adults. If the memory advantage for expectancy-incongruent information is abolished under cognitive load, our ability to successfully engage in social cooperation would be impaired because this type of memory is essential for correcting maladaptive behavior tendencies.

However, it is also possible that cognitive load has *no effect* on memory for expectancy-incongruent behaviors. Remembering expectancy-incongruent information seems to be too important to vanish quickly under conditions of high cognitive load. Cooperation is particularly important in stressful situations. The human cognitive system would be badly designed if it would let go of the most important information under distracting and stressful conditions first. Therefore, the cognitive machinery specialized in categorizing other people are often assumed to be automatic (Klein et al., 2002). The same hypothesis can be based on non-functional, schema-based accounts of memory. According to the schema-copy-plus-tag model, schema-atypical information is encoded and retained in the form of unelaborated tags. This encoding strategy is assumed to be frugal in terms of processing resources, and should remain unaffected by cognitive load (Graesser and Nakamura, 1982). Accordingly, source memory for the face of a cheater is often not due to an enhanced recollection of the specific details of the cheating episode, but instead due to the rough classification of the person as a “cheater” in form of emotional tagging (Bell et al., 2012a). Arguably, these unelaborated emotional tags can be automatically encoded even under conditions of high cognitive load. Consistent with this idea, a demanding secondary task at encoding does not always lead to decreased memory for schema-atypical information, but may even result in a more pronounced schema-atypicality effect in source memory (Ehrenberg and Klauer, 2005). The automatic tagging of expectancy-violating behaviors would allow people to successfully engage in social cooperation even under stressful and distracting conditions.

The present series of experiments was designed to discriminate between these two conflicting hypotheses. The first experiment served to replicate the finding that source memory for the cheating or cooperation of others is enhanced for appearance-incongruent behaviors. To anticipate, an asymmetrical source memory advantage for appearance-incongruent cheating was found. In two further experiments, we examined whether this incongruity advantage would vanish under conditions of increased cognitive load. A fourth study was designed to validate the cognitive-load task by showing that this task does indeed interfere with (general) working-memory resources.

## Experiment 1

Experiment 1 served as a replication of the effects reported by Bell et al. (2012b) with the only difference that female instead of male faces were used as stimuli. We expected to replicate the finding that people guess that trustworthy-looking faces would be associated with cooperation and untrustworthy-looking faces with cheating. Furthermore, we expected that participants would remember appearance-incongruent behaviors better than appearance-congruent behaviors. In most experiments (Suzuki and Suga, 2010; Bell et al., 2012b), this memory advantage was asymmetric in that participants remembered cheating better than cooperation when the partners looked trustworthy, but there was only a non-significant tendency toward remembering cooperation better than cheating when the partners looked untrustworthy. This asymmetry should be particularly pronounced for female faces because they elicit more positive social expectations than male faces, which means that the violation of these positive expectations is particularly salient when female faces are used (Kroneisen and Bell, 2013).

### Method

#### Participants

One hundred and twelve students (73 of whom were female) with a mean age of 23 (*SD* = 5) participated in Experiment 1 (**Table 1**). All participants gave written informed consent in accordance with the Declaration of Helsinki. The present experiments are part of a series of experiments that has been approved by the ethics committee of the Department of Experimental Psychology at Heinrich Heine University Düsseldorf.

**Table 1:** Comparison of age, gender, and justice sensitivity (Schmitt et al., 2005) of Experiment 1 and 2 and Experiment 1 and 3, respectively.

	Age	Gender	Justice Sensitivity
Experiment 1	*M* = 23; *SD* = 5	female = 73 male = 39	*M* = 2.93; *SD* = 0.60
Experiment 2	*M* = 24; *SD* = 5	female = 67 male = 42	*M* = 2.80; *SD* = 0.70
Experiment 3	*M* = 22; *SD* = 5	female = 69 male = 34	—
Comparison of Experiment 1 and 2	*t*(219) = 1.78, *p* = 0.08	χ^2^ (1) = 0.33, *p* = 0.57	*t*(219) = 1.42, *p* = 0.16
Comparison of Experiment 1 and 3	*t*(213) = 1.09, *p* = 0.28	χ^2^ (1) = 0.08, *p* = 0.78	—

#### Materials, Procedure, and Design

The same sequential Prisoner’s Dilemma Game was used as in previous studies (Bell et al., 2012b, 2013). In this game, participants were required to invest money into a joint business with partners whose faces were shown on the screen. Participants played with 20 trustworthy-looking partners and with 20 untrustworthy-looking partners. The faces were randomly drawn from a set of 40 trustworthy-looking and 40 untrustworthy-looking frontal facial photographs of women^1^ with a neutral expression (250 × 375 pixel) from the FERET database (Phillips et al., 1998). In a norming study, the untrustworthy-looking faces had received low trustworthiness ratings (*M* = 2.75, *SD* = 0.24) and the trustworthy-looking faces had received high trustworthiness ratings (*M* = 4.28, *SD* = 0.23) on a scale ranging from 1 to 6. Half of the partners in each condition cooperated and the other half cheated.

**FIGURE 1:**
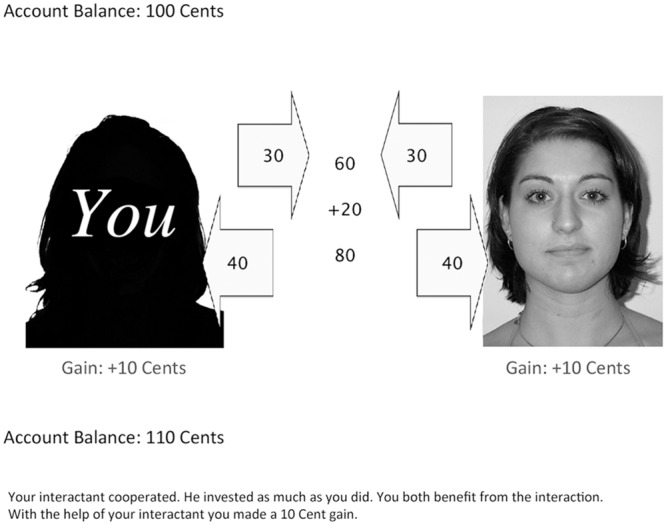
**A screenshot of the sequential prisoner’s dilemma game.** In this example, both the participant and the partner cooperated and invested 30 cents, resulting in a 10 cents gain for each of them. The partner’s photograph shown in this example was taken from the Center for Vital Longevity (CVL) face database (Minear and Park, 2004).

Participants could familiarize themselves with the game in two practice trials. At the start of the game, they were informed that they played for real money. In each trial, participants first saw a silhouette at the left side of the screen (representing the participant), and the partner’s face at the right side of the screen (**Figure 1**). Participants were required to decide whether to invest 15 cents or 30 cents (by pressing a left or right button of the response box, respectively). The decision was displayed on screen for 1 s. The investment was presented in an arrow for 500 ms before it moved to the center of the screen within 500 ms. Similarly, the partner’s decision was shown in an arrow for 500 ms, before it moved to the center of the screen within 500 ms. The sum of investments was then shown in the middle of the screen. After 500 ms a bonus of 1/3 of the sum of investments was added. After 500 ms, the total sum was shown. After a further 500 ms, this total sum was split up between the partners. Both the participant and the partner received half of the total sum, regardless of what they had invested. The partner’s share was shown in an arrow moving toward the partner’s face (500 ms). After 500 ms, the participant’s share was shown in an arrow moving to the participant’s silhouette (500 ms). After 1 s, the partner’s gain or loss was presented, followed by the participant’s gain or loss (after 500 ms). After a further 500 ms, the updated account balance of the participant was presented, and (again after 500 ms) a summary of the interaction was displayed. The next trial was initiated by the participant pressing the continue button.

A cooperating partner always reciprocated the participant’s investment (either 15 or 30 cents), which resulted in a gain for both players. A cheating partner invested nothing (0 cents), which resulted in a gain for the partner at the expense of the participant, who lost money.

The payoff (gain or loss) of each player can be determined by the formula:

$$P_{\text{a}}=\frac{I_{\text{a}}+I_{\text{b}}+\frac{1}{3}\cdot (I_{\text{a}}+I_{\text{b}})}{2}-I_{\text{a}}$$

where *P*_a_ is the payoff of Player A, *I*_a_ is the investment of Player A, and *I*_b_ is the investment of Player B. Applying this formula, it is obvious that interacting with a cooperating partner led to a gain, and interacting with a cheating partner led to a loss of the same magnitude for the participant.

After the game, participants received the instructions for the surprise source memory test. Eighty faces were presented. Half of the faces were old (presented during the sequential Prisoner’s Dilemma Game), and the other half were new. Participants were first required to rate the likability of the faces on a scale ranging from 1 (not likable at all) to 6 (very likable). After pressing the continue button, participants were asked whether or not they had seen the face during the game. If participants indicated that they had seen the face before, they were required to decide whether the face belonged to a cheater or to a cooperator. After pressing the continue button, the next face was shown. Before leaving, participants filled out a paper–pencil version of the justice sensitivity questionnaire (Schmitt et al., 2005), and were paid.

The design was a 2 × 2 repeated measures design with facial trustworthiness (trustworthy vs. untrustworthy) and behavior (cheating vs. cooperation) as independent variables. Dependent variables were game investments, likability ratings, and memory performance. A multinomial model was used to distinguish among old–new recognition, source memory, and guessing processes. Given α = 0.05, a sample size of *N* = 112, and 80 responses in the source memory test, it was possible to detect an effect of size *w* = 0.04 (comparable to the effect sizes observed by Buchner et al., 2009; Küppers and Bayen, 2014; Bell et al., 2015; Kroneisen et al., 2015) for the comparison between source memory for cheaters and cooperators with a statistical power (1 – β) of 0.97. The power calculation was performed using G^∗^Power (Faul et al., 2007).

#### Measuring Source Memory

When examining source memory, it is important to use a measure that does not confound item recognition, source memory, and guessing (Bröder and Meiser, 2007). Therefore, we applied the widely used (Erdfelder et al., 2009) source monitoring model of Bayen et al. (1996) to measure source memory and source guessing separately.

**FIGURE 2:**
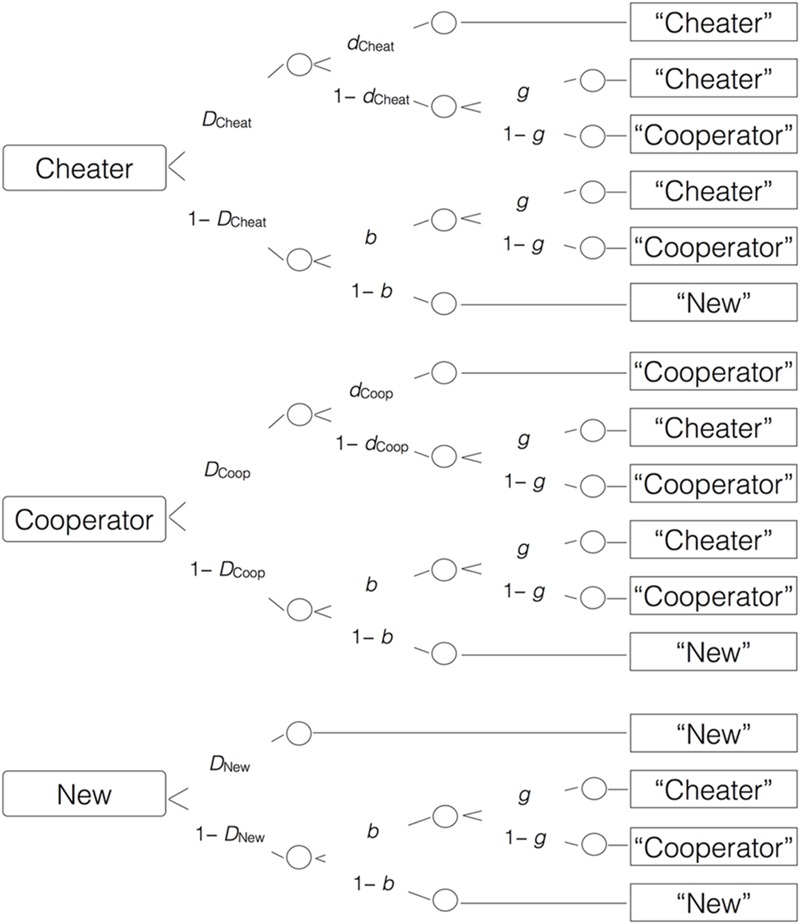
**The multinomial source memory model adapted from Bayen et al. (1996).** Rounded rectangles on the left represent the items presented in the source memory test (cheater, cooperator, or new faces). The letters along the branches represent the probabilities with which certain memory states occur (*D*: probability to correctly recognize a face as old or new; *d*: conditional probability to correctly remember that the person was a cheater or a cooperator; *g*: conditional probability to guess that the person was a cheater; *b*: conditional probability to guess that a face was old). Rectangles on the right represent the participants’ responses in the memory test.

To illustrate, the first model tree in **Figure 2** represents the cognitive states that are assumed to underlie the classification of a cheater face. With probability *D*_Cheat_, participants know that the face is old (remember that they have seen the face during the game). With probability *d*_Cheat_, they also have source memory for the face (remember that the person is a cheater). The source memory parameter is expressed as a conditional probability that varies between 0 and 1. A probability of 0 represents the absence of source memory while a probability of 1 represents perfect source memory. If participants fail to remember the source, which occurs with the complementary probability 1 – *d*_Cheat_, they may guess, with probability *g*, that the person was a cheater or, with probability 1 – *g*, that the person was a cooperator. If they fail to recognize the face as old, which occurs with probability 1 – *D*_Cheat_, they may guess, with probability *b*, that the face is old, and may then guess that the person was a cheater with probability *g*, or that the person was a cooperator with probability 1 – *g*. With probability 1 – *b*, participants may guess that the face is new (has not been encountered during the game). The goodness-of-fit tests are based on the log-likelihood ratio statistic *G*^2^ which is asymptotically chi-square distributed (Riefer and Batchelder, 1988; Stahl and Klauer, 2007; Singmann and Kellen, 2013). Parameter estimations and goodness-of-fit tests were calculated using multiTree (Moshagen, 2010). The observed response frequencies for Experiments 1–3 are reported in the Online Supplementary Material (Data Sheets 1–3).

### Results

#### Game Investments

**FIGURE 3:**
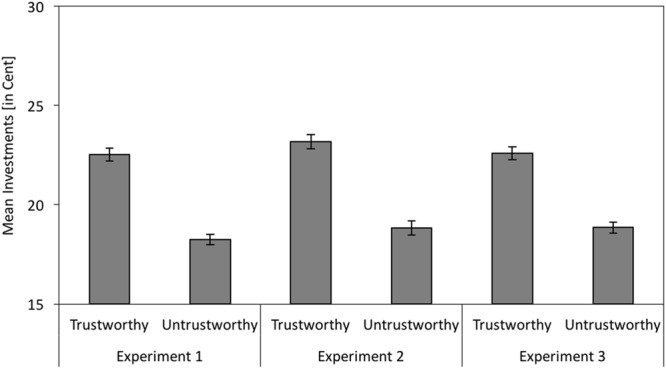
**Participants’ mean investments in the social interaction game as a function of facial trustworthiness (trustworthy vs. untrustworthy) in Experiment 1 (without cognitive load) and in Experiments 2 and 3 (with cognitive load).** The error bars represent the standard errors.

Game investments were analyzed with a repeated measures MANOVA with facial trustworthiness (trustworthy-looking vs. untrustworthy-looking) as independent variable. Participants only interacted once with each partner and thus had no chance to anticipate the behavior of the partners before they decided whether to invest or not. Therefore, only the partners’ facial trustworthiness, but not their behavior could influence the investments. As expected, participants invested more money when playing with trustworthy-looking partners than when playing with untrustworthy-looking partners, *F*(1,111) = 136.83, *p* < 0.001, $\eta_{p}^{2}$ = 0.55 (see left panel of **Figure 3**).

#### Likability Ratings

**FIGURE 4:**
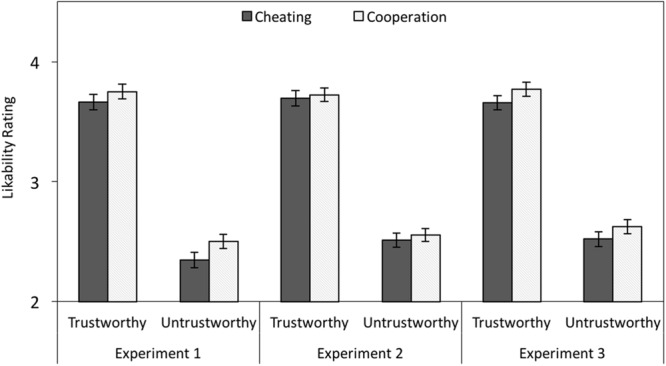
**Mean test-phase likability ratings (on a scale ranging from 1 to 6) as a function of facial trustworthiness (trustworthy vs. untrustworthy) and behavior (cheating vs. cooperation) in Experiment 1 (without cognitive load) and in Experiments 2 and 3 (with cognitive load).** The error bars represent the standard errors.

Likability ratings were analyzed with a 2 × 2 MANOVA with facial trustworthiness (trustworthy-looking vs. untrustworthy-looking) and partner behavior (cheating vs. cooperation) as independent variables. Trustworthy-looking faces were more likable than untrustworthy-looking faces, *F*(1,111) = 410.29, *p* < 0.001, $\eta_{p}^{2}$ = 0.79. Cooperators received higher likability ratings than cheaters, *F*(1,111) = 12.94, *p* < 0.001, $\eta_{p}^{2}$ = 0.10. There was no interaction between facial trustworthiness and behavior, *F*(1,111) = 1.75, *p* = 0.189, $\eta_{p}^{2}$ = 0.01 (see left panel of **Figure 4**).

#### Old–New Recognition

**FIGURE 5:**
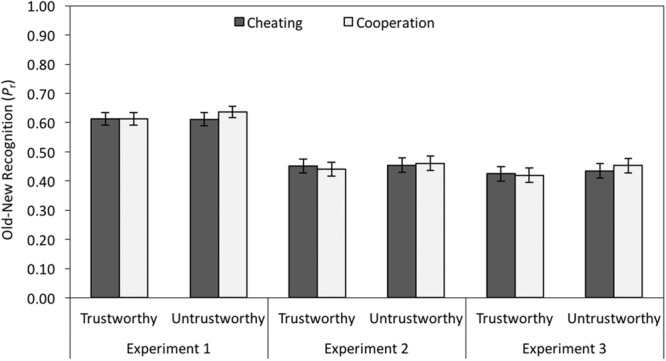
**Old–new recognition in terms of *P*_r_ (corrected hit rates) as a function of facial trustworthiness (trustworthy vs. untrustworthy) and partner behavior (cheating vs. cooperation) in Experiment 1 (without cognitive load) and in Experiments 2 and 3 (with cognitive load).** The error bars represent the standard errors.

Old–new recognition in terms of *P*_r_ (the sensitivity measure of the two-high-threshold model of old–new recognition, often referred to as corrected hit rate and given by hit rate minus false alarm rate; Snodgrass and Corwin, 1988) is shown in the left panel of **Figure 5**. A 2 × 2 MANOVA was performed with facial trustworthiness (trustworthy-looking vs. untrustworthy-looking) and partner behavior (cheating vs. cooperation) as independent variables. There was no main effect of facial trustworthiness on face recognition, *F*(1,111) = 0.52, *p* = 0.472, $\eta_{p}^{2}$ < 0.01, no main effect of partner behavior, *F*(1,111) = 1.11, *p* = 0.294, $\eta_{p}^{2}$ = 0.01, and no interaction between facial trustworthiness and behavior, *F*(1,111) = 0.90, *p* = 0.346, $\eta_{p}^{2}$ < 0.01.

#### Source Guessing and Source Memory

To disentangle source guessing and memory, the multinomial source monitoring model mentioned above (Bayen et al., 1996) was used. For the present study, we needed two sets of the trees displayed in **Figure 2**, one for trustworthy faces and one for untrustworthy faces. To obtain an identifiable base model, we assumed that old–new recognition does not differ as a function of partner behavior (as evidenced by the analysis of old–new recognition reported above), and does not differ between old and new faces (*D*_Cheat_ = *D*_Coop_ = *D*_New_), which is commonly assumed when using the two high threshold model (Snodgrass and Corwin, 1988; Bayen et al., 1996). This base model fit the data well, *G*^2^(2) = 1.84, *p* = 0.398.

**FIGURE 6:**
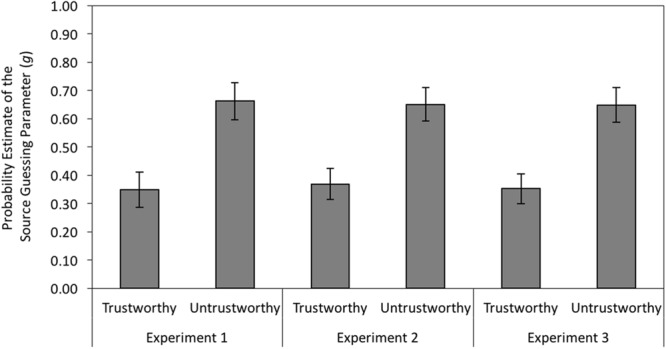
**Estimates of the guessing parameter *g* representing the probability to guess that a person was a cheater rather than a cooperator as a function of facial trustworthiness (trustworthy vs. untrustworthy) in Experiment 1 (without cognitive load) and in Experiments 2 and 3 (with cognitive load).** The error bars represent the 95% confidence intervals.

First, we analyzed whether participants would show an expectancy-congruent guessing bias. When the behavior of a recognized person is not remembered, participants have to guess whether the face was associated with cheating or cooperation. In previous studies (Bell et al., 2012b), participants guessed that trustworthy-looking persons were cooperators and that untrustworthy-looking persons were cheaters. That pattern was replicated here. If source memory was not available at test, participants showed a strong bias toward guessing that trustworthy-looking faces were previously associated with cooperation and that untrustworthy-looking faces were previously associated with cheating, Δ*G*^2^(1) = 43.01, *p* < 0.001, *w* = 0.07 (see left panel of **Figure 6**).

**FIGURE 7:**
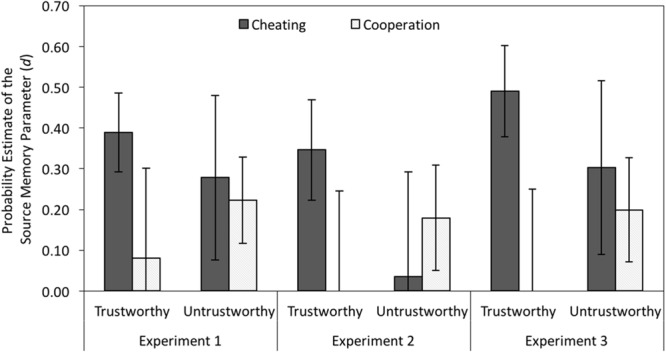
**Estimates of the source memory parameter *d* as a function of the partners’ facial trustworthiness (trustworthy vs. untrustworthy) and the partners’ behavior (cheating vs. cooperation) in Experiment 1 (without cognitive load) and in Experiments 2 and 3 (with cognitive load).** The error bars represent the 95% confidence intervals.

The left panel of **Figure 7** displays the estimates for source memory parameter *d* representing the conditional probability of remembering the behaviors of cheaters and cooperators given that their faces were recognized as old. Source memory was better for cheaters than for cooperators when the faces looked trustworthy, Δ*G*^2^(1) = 4.82, *p* = 0.028, *w* = 0.02, but there was no corresponding memory advantage for cooperators over cheaters when the faces looked untrustworthy, Δ*G*^2^(1) = 0.14, *p* = 0.704, *w* < 0.01. Thus, we replicated the finding of an asymmetrical expectancy-violation effect (Suzuki and Suga, 2010; Bell et al., 2012b).

### Discussion

In Experiment 1, as in previous studies (van ’t Wout and Sanfey, 2008; Bell et al., 2012b, 2013), participants invested more money into the sequential Prisoner’s Dilemma Game (trusted their partners more) when the partners looked trustworthy than when they looked untrustworthy. In the memory test, old–new recognition was not affected by facial trustworthiness and partner behavior, consistent with a large number of previous studies showing that a person’s behavior has no effect on old–new face recognition (e.g., Barclay and Lalumière, 2006; Mehl and Buchner, 2008; Buchner et al., 2009; Kroneisen and Bell, 2013). There are some reports suggesting that old–new recognition is better for untrustworthy-looking than for trustworthy-looking persons (Rule et al., 2012; Bell et al., 2013; Mattarozzi et al., 2015), but this finding was not reliably obtained across experiments (Bell et al., 2012b), and was not replicated here. Consistent with several other studies (Nash et al., 2010; Bell et al., 2012b; Cassidy et al., 2012), participants demonstrated a bias toward guessing that trustworthy-looking persons were cooperators and untrustworthy-looking persons were cheaters. Moreover, and in line with previous studies (Suzuki and Suga, 2010; Bell et al., 2012b), an asymmetric source memory advantage for appearance-incongruent negative information was found: Participants had better source memory for trustworthy-looking cheaters than for trustworthy-looking cooperators.

## Experiment 2

Experiment 2 served to test whether a different pattern of results would be obtained under cognitive load. To impose cognitive load, a continuous choice reaction time (CRT) task with auditory stimuli was used as secondary task. This is a well established method to impose cognitive load (Naveh-Benjamin et al., 2003; Kroneisen et al., 2014), and has the advantage that it involves non-verbal stimuli and responses that do not directly interfere with the sequential Prisoner’s Dilemma Game. Participants had to classify three randomly varying tones by pressing three buttons on a response box. The tones were continuously presented to guarantee a steady burden on cognitive resources. The main question was whether the expectancy-violation effect on source memory would disappear under conditions of reduced cognitive resources.

### Method

#### Participants

One hundred and nine students (67 of whom were female) with a mean age of 24 (*SD* = 5) participated in Experiment 2. Participants in Experiment 2 did not differ from those in Experiment 1 in terms of age, gender, and justice sensitivity (**Table 1**). All participants gave written informed consent.

#### Materials, Procedure, and Design

Experiment 2 was identical to Experiment 1 except that participants were required to perform a secondary CRT task during the sequential Prisoner’s Dilemma Game. The task was to continuously classify three piano tones (C1, F3, and B6) by pressing a black left, gray middle, or white right button on a response box, respectively. Each tone was repeated once every second until participants made a CRT response by pressing a CRT button. Participants received no reminder of the CRT task and no explicit warning when they failed to respond to the CRT stimuli (but the repeated presentation of the same tone can be seen as an implicit warning). Before the start of the sequential Prisoner’s Dilemma Game, participants received a training of the CRT task. During this training, participants received immediate feedback about their responses (“correct” in green font color or “false” or “miss” in red font color). This training continued until participants had 20 correct responses in a row.

Given that participants were not pressured to perform the secondary CRT task, it was necessary to exclude participants who did not respond to the CRT stimuli properly. As an inclusion criterion, we required a minimum of one response per trial in the Prisoner’s Dilemma Game on average. Based on this criterion, datasets of 13 participants were excluded from analyses because of too few CRT responses. With the remaining sample consisting of 96 participants, it was possible to detect an effect of size *w* = 0.04 for the comparison of source memory between cheaters and cooperators with a statistical power (1 – β) of 0.94.

### Results

#### Game Investments

As in Experiment 1, participants invested more when playing with trustworthy-looking partners than when playing with untrustworthy-looking partners, *F*(1,95) = 160.64, *p* < 0.001, $\eta_{p}^{2}$ = 0.63 (see middle panel of **Figure 3**).

#### Likability Ratings

There was a main effect of facial trustworthiness on likability, *F*(1,95) = 433.80, *p* < 0.001, $\eta_{p}^{2}$ = 0.82. The effect of partner behavior was not significant, *F*(1,95) = 1.13, *p* = 0.290, $\eta_{p}^{2}$ = 0.01. There was no interaction between facial trustworthiness and behavior, *F*(1,95) = 0.07, *p* = 0.794, $\eta_{p}^{2}$ < 0.01 (see middle panel of **Figure 4**).

#### Old–New Recognition

Old–new recognition was lower than in Experiment 1, but the same pattern of results was obtained (see middle panel of **Figure 5**). There was neither a main effect of facial trustworthiness, *F*(1,95) = 0.34, *p* = 0.563, $\eta_{p}^{2}$ < 0.01, nor a main effect of partner behavior, *F*(1,95) = 0.02, *p* = 0.897, $\eta_{p}^{2}$ < 0.01. The two-way interaction was not significant, *F*(1,95) = 0.34, *p* = 0.562, $\eta_{p}^{2}$ < 0.01.

#### Source Guessing and Source Memory

The base model fit the data well, *G*^2^(2) = 0.32, *p* = 0.852. As in Experiment 1, participants were more likely to guess that untrustworthy-looking faces were associated with cheating than that trustworthy-looking faces were associated with cheating, Δ*G*^2^(1) = 48.32, *p* < 0.001, *w* = 0.08 (see middle panel of **Figure 6**).

Again, source memory was better for cheating than for cooperation when the faces looked trustworthy, Δ*G*^2^(1) = 5.22, *p* = 0.022, *w* = 0.03, and source memory did not differ between cheating and cooperation when the faces looked untrustworthy, Δ*G*^2^(1) = 0.67, *p* = 0.414, *w* < 0.01 (see middle panel of **Figure 7**).

#### Performance in the Continuous Reaction Time Task

The description of the results is incomplete without an analysis of the performance in the CRT task because it is important to test whether or not the enhanced memory for appearance-incongruent cheating is due to a performance trade-off between the encoding of the faces and the CRT task. Therefore, we performed two 2 × 2 MANOVAs with the partner trustworthiness (trustworthy-looking vs. untrustworthy-looking) and partner behavior (cheating vs. cooperation) as independent variables and the proportion of correct responses and the response times (including only correct responses that occurred after > 100 ms) in the CRT task as dependent variables (**Table 2**). Proportion correct did not differ as a function of facial trustworthiness, *F*(1,95) = 2.43, *p* = 0.122, $\eta_{p}^{2}$ = 0.02. However, CRT performance was less accurate in the cheater condition in comparison to the cooperator condition, *F*(1,95) = 5.76, *p* = 0.018, $\eta_{p}^{2}$ = 0.06. There was no interaction between facial trustworthiness and partner behavior, *F*(1,95) = 0.14, *p* = 0.704, $\eta_{p}^{2}$ < 0.01. Response times showed a similar pattern. Response time did not differ as a function of facial trustworthiness, *F*(1,95) = 0.31, *p* = 0.578, $\eta_{p}^{2}$ < 0.01. Responses were slower in the cheater condition in comparison to the cooperator condition, *F*(1,95) = 5.09, *p* = 0.026, $\eta_{p}^{2}$ = 0.05. However, there was no interaction between facial trustworthiness and partner behavior, *F*(1,95) = 0.15, *p* = 0.697, $\eta_{p}^{2}$ < 0.01. Given that this attentional disruption did not translate into better memory for cheaters (as shown by the analyses above), this result does not seem to reflect a reallocation of cognitive resources to the cheater faces and, therefore, does not seem to reflect a performance trade-off between the memory task and the CRT task. It seems possible to speculate that experiencing cheating may result in a negative emotional response that may distract from the secondary task, but does not seem to cause a direct memory enhancement.

**Table 2:** Mean proportion correct and response times in milliseconds in the CRT task as a function of the partners’ facial trustworthiness (trustworthy vs. untrustworthy) and the partners’ behavior (cheating vs. cooperation) in Experiments 2 and 3.

	Cheating		Cooperation	
	*M*	*SE*	*M*	*SE*
**Experiment 2**				
Proportion correct				
Trustworthy Faces	0.90	0.01	0.92	0.01
Untrustworthy Faces	0.91	0.01	0.92	0.01
Response time				
Trustworthy Faces	2,252	79	2,186	86
Untrustworthy Faces	2,240	99	2,149	86
**Experiment 3**				
Proportion correct				
Trustworthy Faces	0.88	0.01	0.90	0.01
Untrustworthy Faces	0.89	0.01	0.90	0.01
Response time				
Trustworthy Faces	788	13	762	13
Untrustworthy Faces	787	12	760	13

### Discussion

Even though participants had to perform a secondary CRT task, the results were almost identical to those of Experiment 1. Most importantly, participants showed evidence of an appearance-congruent guessing bias and of an asymmetrical expectancy-violation effect on source memory. We conclude from these findings that the enhanced memory for expectancy-incongruent information is obtained even under conditions of cognitive load, which suggests that the encoding of this information occurs automatically and does not rely on demanding elaborative processes.

It seemed important to address the possible concern that the CRT task may simply not have been demanding enough to interfere with the primary task. In Experiment 2, participants were required to perform the secondary CRT task concurrently to the Prisoner’s Dilemma Game, but no time pressure was imposed. Therefore, it may have been possible to attend to both the CRT task and the Prisoner’s Dilemma Game by delaying responses in the CRT task. In Experiment 3, we therefore required participants to respond to each tone within a time interval of 2 s (which is a typical time interval in CRT studies, see Kroneisen et al., 2014).

## Experiment 3

Experiment 3 was identical to Experiment 2 with the exception that the CRT task was modified to increase the continuous demands on cognitive resources.

### Method

#### Participants

One hundred three students (69 of whom were female) with a mean age of 22 (*SD* = 5) participated in Experiment 3. The sample was similar to those in Experiments 1 and 2 (**Table 1**). All participants gave written informed consent.

#### Materials, Procedure, and Design

Experiment 3 was identical to Experiment 2 with the exception that the CRT task required participants to respond to each tone within 2 s, after which the next tone was presented. If participants failed to respond to a tone during a trial of the sequential Prisoner’s Dilemma Game, they received a warning after the trial that reminded them of the CRT task. In contrast to Experiment 2—in which the sequential Prisoner’s Dilemma Game was self-paced—the next round of the game was automatically initiated 10 s after the summary of the interaction had been displayed. Justice sensitivity was not assessed.

The data of two outliers were excluded from the analyses because these participants produced >20% CRT misses on average. The remaining sample responded to 98% of the CRT stimuli on average. With a remaining sample of 101 participants, it was possible to detect an effect of size *w* = 0.04 for the comparison between source memory for cheaters and cooperators with a statistical power (1 – β) of 0.95.

### Results

#### Game Investments

As in Experiments 1 and 2, participants invested more when playing with trustworthy-looking partners than when playing with untrustworthy-looking partners, *F*(1,100) = 157.95, *p* < 0.001, $\eta_{p}^{2}$ = 0.61 (see right panel of **Figure 3**).

#### Likability Ratings

There was a main effect of facial trustworthiness on likability with higher likability ratings for trustworthy-looking compared to untrustworthy-looking partners, *F*(1,100) = 504.95, *p* < 0.001, $\eta_{p}^{2}$ = 0.83. Cheaters were judged to be less likable than cooperators, *F*(1,100) = 15.08, *p* < 0.001, $\eta_{p}^{2}$ = 0.13. The interaction between facial trustworthiness and behavior was not significant, *F*(1,100) = 0.05, *p* = 0.822, $\eta_{p}^{2}$ < 0.01 (see right panel of **Figure 4**).

#### Old–New Recognition

There was neither a main effect of facial trustworthiness on old–new recognition, *F*(1,100) = 1.49, *p* = 0.225, $\eta_{p}^{2}$ = 0.01, nor a main effect of partner behavior, *F*(1,100) = 0.21, *p* = 0.651, $\eta_{p}^{2}$ < 0.01. The two-way interaction was also not significant, *F*(1,100) = 0.57, *p* = 0.452, $\eta_{p}^{2}$ < 0.01 (see right panel of **Figure 5**).

#### Source Guessing and Source Memory

The base model fit the data well, *G*^2^(2) = 0.87, *p* = 0.647. As in Experiments 1 and 2, participants were significantly more likely to guess that untrustworthy-looking faces were associated with cheating than that trustworthy-looking faces were associated with cheating, Δ*G*^2^(1) = 55.78, *p* < 0.001, *w* = 0.08 (see right panel of **Figure 6**).

As in the previous experiments, there was a source memory advantage for cheaters over cooperators when the faces looked trustworthy, Δ*G*^2^(1) = 12.60, *p* < 0.001, *w* = 0.04, but source memory did not differ between cheaters and cooperators when the faces looked untrustworthy, Δ*G*^2^(1) = 0.42, *p* = 0.519, *w* < 0.01 (see right panel of **Figure 7**).

#### Performance in the Continuous Reaction Time Task

As in Experiment 2 we performed analyses of the proportion of correct responses and response times (including only correct responses that occurred after >100 ms) in the CRT task. CRT responses were faster than they were in Experiment 2, but the same pattern of results was observed (**Table 2**). Proportion correct did not differ as a function of facial trustworthiness, *F*(1,100) = 0.42, *p* = 0.520, $\eta_{p}^{2}$ < 0.01. CRT performance was less accurate in the cheater condition in comparison to the cooperator condition, *F*(1,100) = 21.82, *p* < 0.001, $\eta_{p}^{2}$ = 0.18. There was no interaction between facial trustworthiness and partner behavior, *F*(1,100) = 0.55, *p* = 0.460, $\eta_{p}^{2}$ < 0.01. Response times showed a similar pattern. Response time did not differ as a function of facial trustworthiness, *F*(1,100) = 0.09, *p* = 0.764, $\eta_{p}^{2}$ < 0.01. However, responses were slower in the cheater condition in comparison to the cooperator condition, *F*(1,100) = 33.29, *p* < 0.001, $\eta_{p}^{2}$ = 0.25. There was no interaction between facial trustworthiness and partner behavior, *F*(1,100) = 0.04, *p* = 0.845, $\eta_{p}^{2}$ < 0.01. Again, the previous analyses suggest that this attentional disruption is not associated with enhanced encoding of the cheater faces.

### Discussion

Even though participants were pressured to make faster responses in the CRT task, the same pattern of results was obtained as in Experiments 1 and 2. Most importantly, we obtained evidence in favor of an expectancy-congruent guessing bias and of an asymmetric expectancy-violation effect. Therefore, it seems possible to conclude that the encoding of expectancy-incongruent information works well even under conditions of high cognitive load, presumably because it occurs automatically. At a descriptive level, the results of all three experiments are strikingly similar with the only exception that old–new recognition seems to be somewhat decreased in Experiments 2 and 3 in comparison to Experiment 1.

Given that the CRT task did not seem to have any substantial effect on source memory (or any other variable except face recognition), it may be tempting to conclude from these findings that the CRT task was simply not demanding enough. However, concluding from a non-significant finding that the cognitive load manipulation was not strong enough is problematic because this type of circular reasoning renders the prediction that cognitive load affects cooperation and memory unfalsifiable. To escape this problem, we performed a validation study to test whether the secondary task does indeed disrupt cognitively demanding working-memory processes (as intended).

## Experiment 4

Experiment 4 served to validate the CRT task by testing whether it does indeed have the capacity to disrupt cognitively demanding processes. We used both a verbal memory task and a spatial memory task to test whether the CRT task interferes generally with cognitive processing and does not only selectively affect the processing of a specific type of information (Lange, 2005; Vachon et al., in press).

### Method

#### Participants

Forty students (27 of whom were female) with a mean age of 24 (*SD* = 4) participated in Experiment 4. Participants were consecutively assigned to either the cognitive load group or the control group (i.e., Participant 1 was assigned to the cognitive load condition, Participant 2 was assigned to the control condition, and so on). All participants gave written informed consent.

#### Materials, Procedure, and Design

Participants performed a verbal working memory task and a spatial working memory task. Task order was counterbalanced between groups (cognitive load vs. control).

In the verbal working memory task, participants were required to remember sequences with varying sequence lengths of four to nine items. The items were randomly drawn from the set {1, 2, … 9}. Each trial started with a visual warning that participants were required to remember the digits. The digits were presented one after another in 24 pt Arial font at the center of a computer screen for 800 ms with a 200 ms inter-stimulus interval. After a retention interval of 2 s, a number pad with the previously presented digits was shown, and participants were required to select the numbers in the correct (forward) order, using the computer mouse. Selected digits were grayed out, and could not be selected again. After all digits were selected, the number pad disappeared, and a continue button was shown. Upon clicking this button, the next trial started. The task started with a sequence length of four digits. Digit length gradually increased during the task. Participants completed three trials of each sequence length.

The spatial working memory task was identical to the verbal working memory task except that participants were required to remember the spatial locations of four to nine black dots instead of four to nine digits. The locations of the dots were not aligned (but instead randomly distributed across the screen) to make a verbal coding strategy extremely difficult. In each trial, the spatial positions were randomly drawn from a set of nine different spatial positions. The dots appeared one after another at their designated positions (800 ms on, 200 ms off). After a retention interval of 2 s, the previously presented dots were presented again at their corresponding spatial locations. The participants’ task was to select the spatial locations of the dots in the order of their appearance. Selected locations were grayed out, and could not be selected again.

The working memory tasks were either completed alongside the secondary CRT task (in the cognitive load condition) or without the secondary CRT task (in the control condition). The CRT task was identical to the one used in Experiment 3. Participants were reminded of the tone classification task before each trial. Tones were presented only during visual item presentation and the retention interval of the working memory task, but not during recall. If participants did not give a response to all CRT tones, they received a warning when the recall of the items was completed.

The design was a mixed 2 × 2 design with working memory task (verbal vs. spatial) as a within-subject variable and cognitive load (cognitive load vs. control) as a between-subjects variable. The dependent variable was working memory performance according to a strict scoring criterion (only items remembered in their correct serial position were scored as correct). Given α = 0.05, a total sample size of *N* = 40 participants, and an assumed correlation between the levels of the within-subject variable of ρ = 0.50, an effect of size *f* = 0.50 could be detected for the cognitive load variable with a statistical power (1 – β) of 0.95.

### Results

**FIGURE 8:**
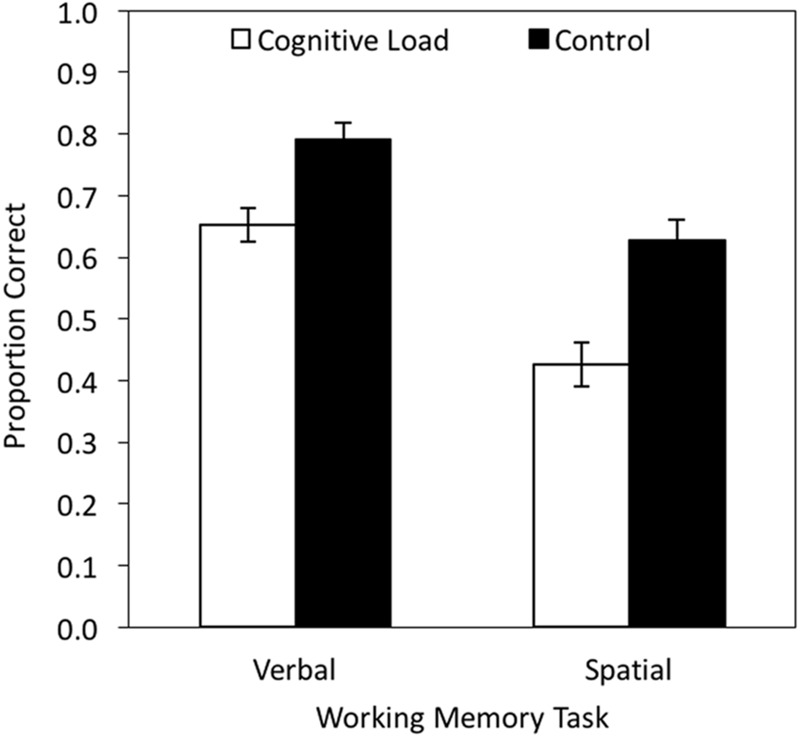
**Mean working memory performance in proportion correct as a function of working memory task (verbal vs. spatial) and cognitive load (cognitive load vs. control).** The error bars represent the standard errors.

A 2 × 2 MANOVA with cognitive load (cognitive load vs. control) and working memory task (verbal vs. spatial) as independent variables yielded a main effect of cognitive load, *F*(1,38) = 20.60, *p* < 0.001, $\eta_{p}^{2}$ = 0.35, and of task, *F*(1,38) = 70.34, *p* < 0.001, $\eta_{p}^{2}$ = 0.65, but no interaction between cognitive load and task, *F*(1,38) = 1.75, *p* = 0.193, $\eta_{p}^{2}$ = 0.04. Cognitive load significantly decreased memory performance both in the verbal, *t*(38) = 3.68, *p* = 0.001, $\eta_{p}^{2}$ = 0.26, and in the spatial task, *t*(38) = 4.05, *p* < 0.001, $\eta_{p}^{2}$ = 0.30 (**Figure 8**). Raw data are reported in the Online Supplementary Material (Data Sheet 4).

### Discussion

Experiment 4 serves as a validation study to confirm that the CRT task interferes with cognitively demanding processes. In line with our expectations, the CRT task disrupted performance in a verbal working memory task as well as in a spatial working memory task, suggesting that it does not only interfere with a specific type of information processing, but instead leads to a general decrease of cognitive resources. This rules out the possibility that the CRT task was not demanding enough to disrupt cognitive processing, which facilitates the interpretation of the findings obtained in Experiments 1–3.

## General Discussion

Previous research suggests that expectations about other people’s trustworthiness are formed quickly and automatically on the basis of physical appearance (Todorov et al., 2009, 2015). Trustworthiness judgments in particular are strongly affected by facial cues (Todorov, 2008). The assumption that facial cues have a strong effect on trust and social expectations (van ’t Wout and Sanfey, 2008) is further confirmed by the present results. Specifically, participants invested more into the sequential Prisoner’s Dilemma Game when the partners looked trustworthy than when the partners looked untrustworthy. Given that investing into the game only payed off when the partner reciprocated, this result suggests that trustworthy-looking partners were expected to cooperate more than untrustworthy-looking partners. Noticeably, this pattern of results was obtained without and with cognitive load, which confirms previous findings suggesting that the perception of facial trustworthiness is an automatic process that does not depend on the availability of cognitive resources (Bonnefon et al., 2013).

Given that appearance-based judgments about a person are often invalid (Todorov et al., 2015), it is important to update facial trustworthiness judgments with behavioral information (Rezlescu et al., 2012). It may be especially important to remember expectancy-incongruent behaviors to be able to correct a false first impression about another person. Consistent with previous studies (Suzuki and Suga, 2010; Volstorf et al., 2011; Bell et al., 2012b), source memory was better for the appearance-incongruent cheating of a trustworthy-looking person in comparison to the appearance-congruent cooperation of a trustworthy-looking person. Noticeably, memory for appearance-congruent cooperation was poor. This confirms the predictions of the schema-copy-plus-tag model (Graesser and Nakamura, 1982), which states that discriminability of schema-consistent information is poor because it will be produced at test regardless of whether it was presented at encoding or not. Schema-atypical information is more distinct, and, therefore, associated with better memory discriminability.

Memory was selectively enhanced for cheating that violated a positive expectation about a trustworthy-looking partner, but there was no similar memory advantage for cooperators over cheaters when the faces looked untrustworthy. This asymmetry was also found in previous memory experiments (Suzuki and Suga, 2010; Bell and Buchner, 2012), and it fits with a study on investments in repeated game interactions showing that participants tend to adjust their own behavior more strongly in response to a partner’s defection than in response to a partner’s cooperation (Chang et al., 2010). This asymmetric memory advantage for appearance-incongruent cheating over appearance-incongruent cooperation may be particularly pronounced in the present study because only female stimulus faces were used. It is known that female faces tend to elicit positive social expectations (Kroneisen and Bell, 2013), which means that norm-violating behaviors of female partners may represent particularly strong expectancy violations (Bell et al., 2015).

Two explanations for the memory advantage for appearance-incongruent cheating were tested. According to the first account, information that does not fit into existing schemas receives more elaborative processing, which depends on the mobilization and availability of additional cognitive resources. This enhanced elaboration results in a more vivid and detailed recollection of the expectancy-incongruent information. According to the second account, schema-atypical information is retained in form of unelaborated tags. This resource-efficient encoding strategy has the advantage that unexpected information can be encoded and retained in memory even under conditions of high cognitive load. The present results support the latter view. The source memory advantage for appearance-incongruent cheating was not affected by the presence or absence of cognitive load at encoding. A similar memory advantage for appearance-incongruent cheating was obtained in all three experiments, regardless of whether participants had to perform a demanding secondary task at encoding or not. The experiments were reported separately because they were run at different times. However, when the source memory data of all experiments were combined in a single supplementary cross-experimental analysis, the conclusion that source memory was not affected by cognitive load was supported. The base model still fit the data well, *G*^2^(6) = 3.02, *p* = 0.807. Source memory did not differ among experiments, Δ*G*^2^(8) = 11.95, *p* = 0.154, *w* = 0.02, which suggests that the pattern of results was not affected by the secondary task in Experiments 2 and 3.

This pattern of findings confirms the predictions of the schema-copy-plus-tag model (Graesser and Nakamura, 1982), according to which schema-violating information is retained in the form of simple tags that require only minimal elaboration, and can therefore be encoded and retained even under conditions of high cognitive load. Consistent with this interpretation, it has been previously shown that the source memory advantage for faces of cheaters is not due to a vivid recollection of the cheating episode, but rather due to emotional tagging in the sense of a rough classification of the partner as a “cheater” (Bell et al., 2012a). The encoding and retrieval of simple emotional tags may be less cognitively demanding and, therefore, less affected by a reduction in cognitive resources than other types of context memory (Rahhal et al., 2002).

This interpretation fits well with Todorov and Uleman’s (2003) assumption that reading about or observing the behavior of another person leads people to draw inferences about the other person’s traits (e.g., dishonest or honest) that then become linked to the other person’s face. Importantly, these trait representations are assumed to include only a summary judgment about the other person’s behavior, and to be comparatively unelaborated and robust (Carlston and Skowronski, 1994; Todorov and Uleman, 2002). In the study of Todorov and Uleman (2003), participants saw faces with behavior descriptions that implied character traits. The binding between faces and traits was revealed by an enhanced false recognition of the trait labels in an implicit memory test. The most interesting finding in the present context is that the implicit memory for the association between a face and a trait was not affected by a secondary task at encoding (rehearsing 6-digit numbers), which suggests that the process of binding traits to faces is an automatic process. The present study shows parallel findings in a different paradigm where traits are directly inferred from experiences in a social-dilemma game, and memory is tested in an explicit source memory test.

Remembering appearance-congruent cooperation and cheating enables participants to update their impressions about other people, which could have beneficial effects on future social decision making. For instance, when we encounter a trustworthy-looking person, but learn subsequently that this person is not to be trusted, memory for the appearance-incongruent cheating may help to avoid being fooled by the trustworthy appearance of this person again. Obviously, this discussion implies that the memory for the partners’ previous behaviors is used to inform social decision making. Previous results using repeated social-dilemma games suggest that people continue to rely on facial trustworthiness over the course of the game (in line with the persistent effect of facial trustworthiness on source guessing in the present experiment), but also succeed in adjusting their own decisions to the individual partners’ previous trustworthy or untrustworthy behaviors (Chang et al., 2010; Rezlescu et al., 2012). Murty et al. (2016) directly examined the relationship between memory and economic decision making, and found that source memory (in contrast to item memory) had a beneficial effect on the participants’ choices in social and non-social decision making tasks. Therefore, it seems plausible to assume that source memory for appearance-incongruent behaviors can have direct beneficial effects on social decision making.

## Conclusion

In sum, source memory for cheaters and cooperators was highly similar across experiments, regardless of whether cognitive load was induced at encoding (Experiments 2 and 3) or not (Experiment 1). These results are compatible with the general idea that cognitive mechanisms underlying social cooperation operate highly automatically so that they remain unaffected by cognitive load. Specifically, it seems possible to encode and retain information about a person’s expectancy-incongruent behavior even under conditions of high cognitive load. Remembering this type of behavior seems particularly important for the decision making process because it helps to correct maladaptive behavior tendencies. For example, it seems particularly important to remember that a trustworthy-looking person is in fact not to be trusted to avoid being fooled by the trustworthy appearance of this person in the future. Being able to remember appearance-incongruent behaviors even under conditions of cognitive load may be beneficial in that it allows people to sustain successful reciprocal cooperation even under the distracting and stressful conditions that are characteristic of everyday life.

## Author Contributions

LM, RB, and AB conceived and designed the experiments, supervised data collection, analyzed the data, and wrote the paper.

## Conflict of Interest Statement

The authors declare that the research was conducted in the absence of any commercial or financial relationships that could be construed as a potential conflict of interest.

## Footnotes

1 As in our previous studies (e.g., Bell et al., 2015) we only used faces from one gender because it is well known that female faces are more trustworthy than male faces (Kroneisen and Bell, 2013), and we did not want facial gender to dilute the facial trustworthiness manipulation.
