# Supplementary material for: Cognitive Load Does Not Affect the Behavioral and Cognitive Foundations of Social Cooperation
Source: Front Psychol. 2016 Aug 31;7:1312. doi: 10.3389/fpsyg.2016.01312 (PMC5006039; doi:10.3389/fpsyg.2016.01312)
Supplement: Supplementary file 1 [file Data_Sheet_1.DOCX]

multinomial Data Experiment 1 (HighTrustworthy Cheaters - HighTrustworthy Cooperators - HighTrustworthy New - LowTrustworthy Cheaters - LowTrustworthy Cooperators - LowTrustworthy New)

1 451

2 344

3 325

4 259

5 537

6 324

7 76

8 142

9 2022

10 584

11 201

12 335

13 434

14 380

15 306

16 133

17 68

18 2039

===
