# Supplementary material for: Cognitive Load Does Not Affect the Behavioral and Cognitive Foundations of Social Cooperation
Source: Front Psychol. 2016 Aug 31;7:1312. doi: 10.3389/fpsyg.2016.01312 (PMC5006039; doi:10.3389/fpsyg.2016.01312)
Supplement: Supplementary file 2 [file Data_Sheet_2.DOCX]

multinomial Data Experiment 2 (HighTrustworthy Cheaters - HighTrustworthy Cooperators - HighTrustworthy New - LowTrustworthy Cheaters - LowTrustworthy Cooperators - LowTrustworthy New)

1 307

2 270

3 383

4 210

5 357

6 393

7 105

8 183

9 1632

10 369

11 190

12 401

13 316

14 249

15 395

16 160

17 86

18 1674

===
