# Supplementary material for: Cognitive Load Does Not Affect the Behavioral and Cognitive Foundations of Social Cooperation
Source: Front Psychol. 2016 Aug 31;7:1312. doi: 10.3389/fpsyg.2016.01312 (PMC5006039; doi:10.3389/fpsyg.2016.01312)
Supplement: Supplementary file 3 [file Data_Sheet_3.DOCX]

multinomial Data Experiment 3 (HighTrustworthy Cheaters - HighTrustworthy Cooperators - HighTrustworthy New - LowTrustworthy Cheaters - LowTrustworthy Cooperators - LowTrustworthy New)

1 341

2 241

3 428

4 206

5 371

6 433

7 105

8 201

9 1714

10 408

11 149

12 453

13 314

14 261

15 435

16 153

17 83

18 1784

===
